# Retraction: Neutrophil extracellular traps promote peritoneal metastasis of colon cancer cells

**DOI:** 10.18632/oncotarget.27482

**Published:** 2020-02-11

**Authors:** Amr A. Al-Haidari, Nader Algethami, Mattias Lepsenyi, Milladur Rahman, Ingvar Syk, Henrik Thorlacius

**Affiliations:** ^1^ Department of Clinical Sciences, Malmö, Section for Surgery, Lund University, 20502 Malmö, Sweden

**This article has been retracted:** This paper is retracted because as of this writing, the authors are not able to correct an erroneous negative control in Figure 6B with an original image. Nevertheless, the conclusions of the paper are correct as when the concern was raised after publication, the authors repeated the experiments and confirmed the original results. The authors are deeply sorry for the error.

Original article: Oncotarget. 2019; 10:1238–1249. 1238-1249. https://doi.org/10.18632/oncotarget.26664

